# *In vitro* exposure to benzo[a]pyrene damages the developing mouse ovary

**DOI:** 10.1530/RAF-22-0071

**Published:** 2023-04-13

**Authors:** Agnes Stefansdottir, Magda Marečková, Magdalena Matkovic, Caroline M Allen, Norah Spears

**Affiliations:** 1 Biomedical Sciences, University of Edinburgh, Edinburgh, UK; 2 School of Life Sciences, University of Glasgow, Glasgow, UK

**Keywords:** benzo[a]pyrene, ovary, follicle, in vitro, germ cell

## Abstract

Females are born with a finite number of oocytes, collectively termed the ovarian reserve, established within the developing fetal ovary. Consequently, maternal exposure to reproductive toxicants can have harmful effects on the future fertility of her unborn female fetus. The chemical benzo[a]pyrene (B[a]P) is a prominent component of cigarette smoke. Despite it being a known ovotoxicant, around 8% of women in Europe smoke during pregnancy.

The purpose of this research was to examine the effect of B[a]P on the developing ovary, using the mouse as a model and with experiments carried out *in vitro*. B[a]P-exposure to the fetal ovary prior to follicle formation reduced the number of germ cells and subsequently, the number of healthy primordial follicles, by up to 76%; however, while proliferation of germ cells was not affected, the germ cells contained higher levels of DNA double-strand breaks. Exposure to B[a]P also affected the proportion of oocytes progressing through prophase I of meiosis. B[a]P exposure to neonatal mouse ovaries, after follicle formation, resulted in an 85% reduction in the number of healthy follicles, with a corresponding increase in apoptotic cell death and reduction in somatic cell proliferation. Although there was a trend towards a higher level of oxidative stress in B[a]P-exposed ovaries, this was not statistically significant; likewise, the antioxidant melatonin failed to protect against the B[a]P-induced ovarian damage. Together, the results here demonstrate that B[a]P-exposure damages the developing ovary, both before and shortly after follicle formation, an effect that could lead to a subsequent decrease in fertility.

**Lay summary:**

Cigarette smoking during pregnancy can affect the fertility of the offspring, yet in Europe around 1 in 12 children born have been exposed to cigarette smoke before birth due to their mother smoking. Benzo[a]pyrene (B[a]P), one of the main chemicals found in cigarette smoke, can have damaging effects on the ovary as it develops in the fetus during the time that the population of future eggs, known as ovarian germ cells also develop. In this research, ovaries from mouse fetuses at stages of development, equivalent to the second and third trimesters of a human pregnancy, were cultured with or without B[a]P. Fetal mouse ovaries exposed to B[a]P had fewer germ cells and larger numbers of cells did not survive. Overall, the results suggest that development of the ovary of a fetus could be affected if the mother is exposed to B[a]P, whether that is through cigarette smoke, or other types of exposure.

## Introduction

Cigarette smoke is a known reproductive hazard that can affect fertility in female smokers. It has been associated with a reduction in the ovarian reserve, premature ovarian insufficiency and reduced pregnancy rates in women undergoing IVF (El-Nemr *et al.* 1998, Shiloh *et al.* 2004, Neal *et al.* 2005, Freour *et al.* 2008, Neal *et al.* 2008). Women who smoke have been found to undergo menopause on average a year earlier than women who refrain from smoking (Harlow & Signorello 2000). In addition, cigarette smoking during pregnancy has well-known effects on fetal developmental outcomes: these include an increased risk of fetal growth restriction, preterm birth and sudden infant death syndrome (DiFranza & Lew 1995, Haustein 1999, Harvey *et al.* 2007). Given its reproductive toxicity, it is not surprising that cigarette smoke has also been shown to have damaging effects on the fertility of both male and female offspring that have been exposed to maternal smoke during fetal life (Storgaard *et al.* 2003, Coutts *et al.* 2007, Fowler *et al.* 2009, Lutterodt *et al.* 2009, Mamsen *et al.* 2010, Ernst *et al.* 2012, Anderson *et al.* 2014, Fowler *et al.* 2014). Specifically, in both the human and the mouse model, prenatal exposure of the female fetus to cigarette smoke has been shown to lead to premature depletion of ovarian germ cells, a reduction in the number of ovarian somatic cells and impaired fertility (MacKenzie & Angevine 1981, Vähäkangas *et al.* 1985, Lutterodt *et al.* 2009, Mamsen *et al.* 2010, Fowler *et al.* 2014, Camlin *et al.* 2016, Luderer *et al.* 2019, Rahmani *et al.* 2021). Despite the aforementioned risks to the fetus, it is estimated that up to 8% of women in Europe smoke cigarettes throughout their pregnancy (NHS UK 2023, Lange *et al.* 2018, Lifestyles Team 2021). Fewer than 4% of pregnant women subsequently quit smoking (Tappin *et al.* 2010, Observatory SPH 2019, Digital 2021).

Cigarette smoke is composed of over 4000 different chemicals (Richter *et al.* 2008), many of which can have harmful effects on the developing ovary. Of these, one of the components on which there is the most evidence of toxicological effects is benzo[a]pyrene (B[a]P). B[a]P is a polycyclic aromatic hydrocarbon (PAH) that is found in high levels in the tar of cigarette smoke. Non-smokers are also exposed to B[a]P, through chargrilled foods, wood smoke, coal and roofing tar, vehicle exhaust and haze (Lijinsky 1991, Rodgman *et al.* 2000, Chang *et al.* 2006, EEA 2017, Cao *et al.* 2018). PAHs are mutagenic and carcinogenic chemicals that can be inhaled or absorbed through the skin, as well as being readily absorbed from the gastrointestinal tract, resulting in a rapid distribution throughout the body (Samanta 2002, IARC 2010). PAHs, including B[a]P, are able to cross the placenta and enter the fetal bloodstream (Machado *et al.* 2015), but there is limited information about the amount of B[a]P that reaches the developing fetus.

PAHs can exert their toxic effect on the ovary through binding to the aryl hydrocarbon receptor (AhR), with AhR expressed in both mouse and human oocytes (Robles *et al.* 2000, Anderson *et al.* 2014). Activation of AhR in the human fetal ovary results in a reduction of germ cell proliferation (Anderson *et al.* 2014). There is also evidence to suggest that B[a]P may induce cytotoxicity through the production of reactive oxygen species (ROS), leading to oxidative stress: this can compromise the meiotic progression in maturing oocytes (Sobinoff *et al.* 2012, Sobinoff *et al.* 2013, Miao *et al.* 2018, Zhang *et al.* 2018). Indeed, the antioxidant hormone N-acetyl-5-methoxytryptamine (melatonin) has been shown to protect cells against oxidative stress and apoptosis (Tamura *et al.* 2008, Tamura *et al.* 2012). Melatonin has also been linked to the regulation of ovarian function and it has been shown to act as an antioxidant to reduce oxidative stress during oocyte maturation and embryo development (Bahadori *et al.* 2013, Reiter *et al.* 2014, Rodrigues-Cunha *et al.* 2016). Melatonin also protects B[a]P-exposed oocytes from meiotic failure (Miao *et al.* 2018). It is, however, not known whether melatonin can ameliorate B[a]P-induced damage to the developing ovary.

**Figure 1:**
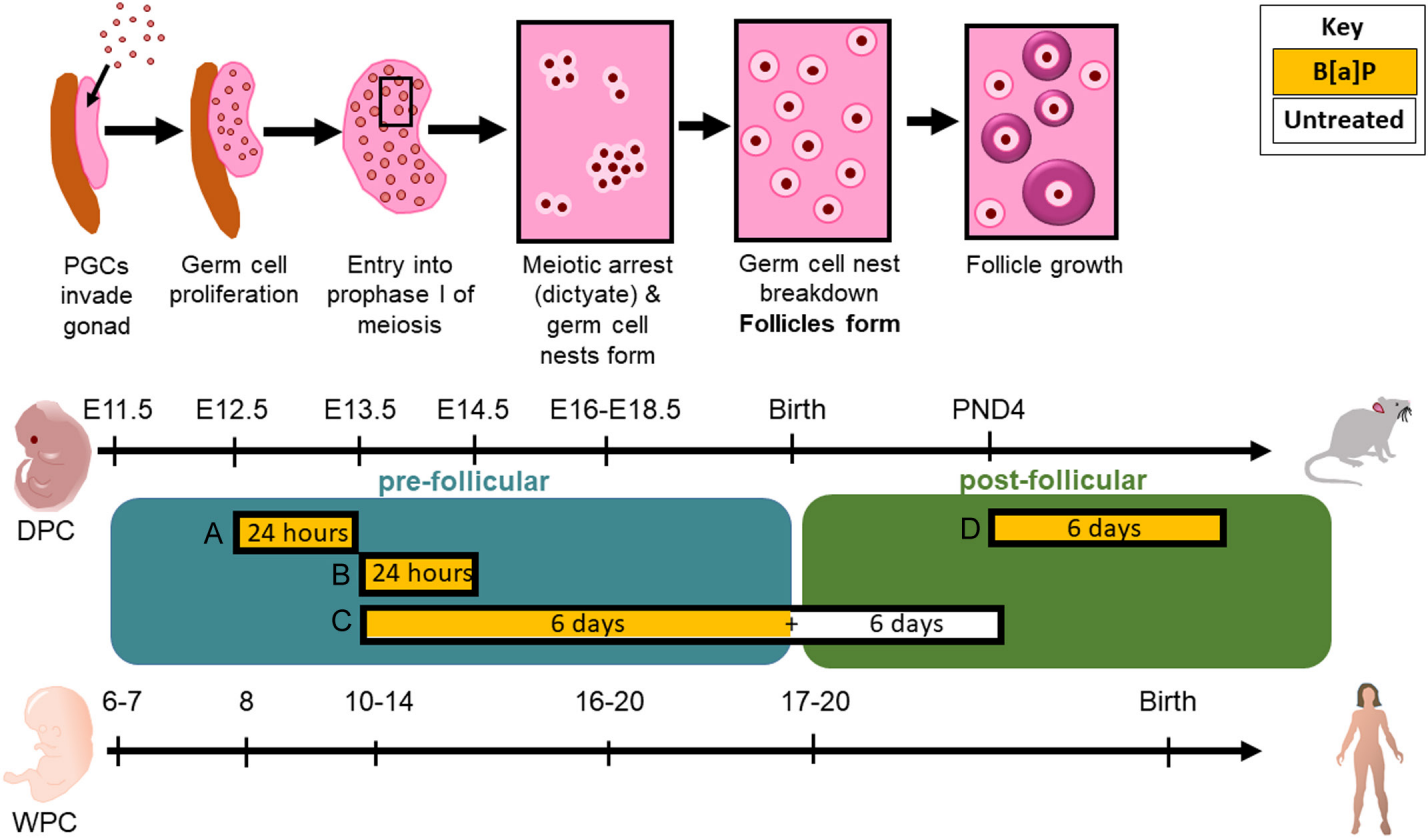
Comparison of timings of developmental events between the mouse and human ovary. Migrating PGCs invade the developing female gonad, after which time the germ cells continue to proliferate until they enter into prophase I of the first meiotic division. Oogonia undergo early stages of prophase I, at which point germ cell nests have begun to form. Subsequently, the germ cell nests begin to break down, with follicles forming around the time of birth in the mouse, but during the second trimester of the human pregnancy. From the point of PMF formation, some PMFs will gradually begin to initiate follicle growth. Follicle growth starts only after birth in the mouse ovary, but from the second and third trimesters in the human ovary. Black boxes indicate culture periods used in the current study with yellow areas indicating the duration of B[a]P administration, and white areas indicating the period of time where ovaries were kept in untreated medium: (A) E12.5 ovaries cultured for 24 h with B[a]P or DMSO, (B) E13.5 ovaries cultured for 24 h with B[a]P or DMSO, (C) E13.5 ovaries cultured for 12 days with B[a]P or DMSO added to the media for the first 6 days of culture only and left in the untreated medium for a further 6 days and (D) PND4 ovaries cultured for 6 days with B[a]P or DMSO. DPC, days post-coitum; WPC, weeks post-conception.

Female germ cells begin to form in the developing human fetal gonad during the first trimester of pregnancy (Baker & Zuckerman 1963, Eddy *et al.* 1981). After an initial phase of rapid cell proliferation, during which the germ cells are termed oogonia, the cells enter the first prophase I of the first meiotic division around the 13th week of gestation and then progress through the first meiotic division up to the diplotene stage of prophase I of meiosis. At this point, the germ cells, now termed oocytes, enter meiotic arrest and become surrounded by pre-granulosa cells, together forming primordial follicles (PMFs). Follicles begin to form as early as the 17th week of gestation, although they can continue to form and initiate growth until late pregnancy (Forabosco & Sforza 2007). By comparison, in the developing mouse ovary, oogonia enter meiosis around embryonic day 13.5 (E13.5), with the now oocytes reaching meiotic arrest by E19.5, around the end of gestation, and with follicle formation occurring around the time of birth (Fig. 1).

A pregnant woman can be exposed to B[a]P at any point during gestation: consequently, any female fetus can be exposed both before and after follicle formation. Given this situation, it is important to investigate not only how B[a]P affects the ovary once PMFs have formed but also how B[a]P can affect earlier stages of germ cell development, prior to PMF formation. Any effect on germ cell proliferation, prophase I of meiosis or germ cell nest breakdown is likely to affect subsequent PMF formation and consequently, the ovarian reserve. To date, the majority of studies have concentrated on the effect of B[a]P at later stages of ovarian follicle development, once the ovarian follicle pool has already been established (Swartz & Mattison 1985, Mattison *et al.* 1989, Miller *et al.* 1992, Borman *et al.* 2000, Neal *et al.* 2007, Neal *et al.* 2010, Sadeu & Foster 2011, Archibong *et al.* 2012, Sobinoff *et al.* 2012, Sadeu & Foster 2013, Einaudi *et al.* 2014, Zhang *et al.* 2018). In contrast, few studies have examined its effects on earlier stages of ovarian development, representative of human fetal ovary exposure (MacKenzie & Angevine 1981, Kristensen *et al.* 1995, Lim *et al.* 2016, Luderer *et al.* 2017, Luderer *et al.* 2019, Rahmani *et al.* 2021). As a result, it is still not certain how maternal B[a]P exposure affects these key early ovarian developmental events.

The aim of this study was to investigate how B[a]P exposure might affect the human fetal ovary, with experiments using the mouse as a model. Two ovary culture systems were used, in order to investigate the effect of B[a]P exposure during the two distinct stages of human fetal ovary development (Stefansdottir *et al.* 2016) (Fig. 1). The first stage spans the developmental events leading up to the time of follicle formation, including germ cell proliferation, germ cell nest breakdown and progression through prophase I of meiosis to the diplotene stage. The second stage covers when germ cells are contained within ovarian follicles, with that follicular environment potentially offering the oocytes increased protection (Stefansdottir *et al.* 2016). Using the mouse ovary as a model, we have examined whether ovarian exposure to B[a]P impacts the number or health of germ cells/follicles prior to and following follicle formation. Finally, we have examined whether B[a]P leads to the production of ROS in the developing ovary and whether the antioxidant melatonin can protect against any oxidative stress.

## Methods

### Animals

All work was approved by the University of Edinburgh’s Local Ethical Review Committee and carried out in accordance with UK Home Office regulations. Wild-type CD-1 mice were maintained and bred in an environmentally controlled room on a 14 h light:10 h darkness photoperiod. To obtain fetuses for fetal ovary culture experiments, mouse breeding harems were set up and females were checked for the presence of a copulation plug, then designated as E0.5.

### Ovary culture

#### Fetal mouse ovary culture: pre-follicular exposure

Pregnant timed-mated females were obtained at E12.5 or E13.5 and culled by cervical dislocation. Mouse fetuses were dissected out of the uterus, removed from their amniotic sac and the placenta removed. Fetuses were placed in a petri dish containing 1× PBS at 4°C and then decapitated. A ventral incision was made in the fetus to remove the heart, bowels and liver from the peritoneal cavity. Fetal ovaries and the attached mesonephroi were dissected from any females, placed on a 2% agar block and cultured in a 33-mm petri dish (Sigma Aldrich Ltd) incubated at 37°C, 5% CO_2_, for 24 h, 72 h or 12 days, as previously described (Stefansdottir *et al.* 2016).

The culture medium used for the first 3 days of culture (days 0–3), consisted of Dulbecco’s Minimal Essential medium (Life Technologies) supplemented with 10% fetal calf serum (Thermo Fisher), 2 mM l-glutamine (Invitrogen), 10 μM β-mercaptoethanol (Life Technologies), 1% sodium pyruvate (Sigma Aldrich Ltd), 1% penicillin/streptomycin (Invitrogen) and 1% amphotericin B (Sigma Aldrich Ltd). From day 3 onwards, this culture medium was replaced with a simpler culture medium composed of αMEM medium (Invitrogen) supplemented solely with 3 mg/mL bovine serum albumin (Sigma Aldrich Ltd).

The medium was topped up daily as required in order to keep the level of medium in line with the top of the agar block to prevent the tissue from drying out. In addition, every 48 h, half of the medium (approximately 1.5 mL) was removed and replenished with fresh medium.

The effect of B[a]P on ovary development was examined by adding increasing doses of B[a]P (Sigma Aldrich Ltd) to produce final concentrations of 0.01, 0.1or 1 µg/mL for the first 6 days of culture (12-day culture). B[a]P was dissolved in dimethylsulphoxide (DMSO) (Sigma Aldrich Ltd); therefore, DMSO was also added to the control medium, with all media containing 0.1% DMSO. Cultures were maintained for 24 h, 72 h or 12 days. For the shorter duration cultures (24-h and 72-h cultures), only the top dose (1 µg/mL) of B[a]P was used. For the 24-h ovary cultures, the medium was additionally supplemented with 15 µg/mL bromodeoxyuridine (BrdU), a thymidine analogue that is incorporated into replicating DNA, for determination of germ cell proliferation (Sigma Aldrich Ltd). For the 12-day cultures, ovaries were transferred to a drug-free culture medium on day 6 of culture and maintained in that medium for a further 6 days of culture. In this culture system, follicles begin to form from around day 6 onwards (Stefansdottir *et al.* 2016). Therefore, B[a]P exposure was removed from day 6 of culture onwards in order to limit the window of B[a]P exposure to the developmental period leading up to follicle formation.

#### Neonatal mouse ovary culture: post-follicular exposure

Post-natal day 4 (PND4) female mice were culled by decapitation and ovaries were dissected into Leibovitz dissection medium (Invitrogen) supplemented with 3 mg/mL bovine serum albumin (Sigma Aldrich). Ovaries were cultured for 6 days in a 24-well culture plate (Greiner Bio-one, Stonehouse, UK) on 13-mm 8.0-µm Whatman nucleopore polycarbonate membranes (Camlab Ltd, Cambridge, UK) incubated at 37°C, 5% CO_2_ as described previously (Morgan *et al.* 2013). Culture medium consisted of αMEM medium (Invitrogen) supplemented with 3 mg/mL bovine serum albumin. Half of the culture medium (500 µL) was exchanged with fresh medium every other day. The medium was supplemented with increasing doses of B[a]P to produce final concentrations of 0.01, 0.1 or 1 µg/mL for the duration of culture. B[a]P was dissolved in DMSO, which was therefore also added to the control medium, with all media containing 0.1% DMSO.

In order to investigate cell proliferation in neonatal ovaries, PND4 mouse ovaries were cultured as above, in the presence or absence of B[a]P (1 µg/mL). The medium was additionally supplemented with 15 µg/mL BrdU (Sigma Aldrich Ltd) for the last 24 h of culture. Here, paired ovaries were used, with one ovary from each embryo used as a control and the other paired ovary cultured with 1 µg/mLB[a]P. For cultures containing melatonin (Sigma Aldrich Ltd, dissolved in DMSO), melatonin was added to the culture medium to produce a final concentration of 10 µg/mL for the duration of the neonatal culture (6 days). This concentration of melatonin was chosen after carrying out an initial dose–response experiment using a range of melatonin concentrations, based on the highest dose tested that did not show a detrimental impact on follicle health, since at melatonin concentrations higher than 10 µg/mL, there was a negative effect on follicle health (data not shown).

### Fixation, processing and histological follicle assessment

At the end of culture, ovaries were placed in 10% neutral-buffered formalin solution (Sigma Aldrich Ltd) for 24 h at room temperature, washed in 70% ethanol, set in 3% agar (Sigma Aldrich Ltd), processed and embedded in paraffin wax.

Wax blocks were sectioned at 5 µm and stained with haemotoxylin and eosin. For fetal ovaries, every 6th section, and for neonatal ovaries, every 12th section was photomicrographed (DMLB Leica microscope, Leica Microsystems Ltd), and follicles were counted using Image J (National Institute for Health, Bethesda, MD, USA). Follicle developmental stage and health were classified. A follicle was considered to be at the PMF stage if only flattened pre-granulosa cells were visible, at the transitional stage (TRNS) if it contained both flattened and cuboidal granulosa cells or at the primary (PRMRY) or secondary (SEC) stage when the oocyte was visibly larger with one or two complete layer(s) of cuboidal granulosa cells, respectively. Follicles were considered healthy if they contained a round oocyte with a central nucleus and evenly stained cytoplasm, along with the complete absence of pyknotic granulosa cells: in contrast, oocytes were considered unhealthy if they were misshapen, or they contained a shrunken or pyknotic nucleus, while granulosa cells were identified by dark eosin staining. All analysis was carried out with an assessor blind as to treatment. In order to estimate total follicle numbers, counts were corrected using the Abercrombie correction factor (Abercrombie 1946). For histological examination of follicle numbers and health in cultured fetal and neonatal ovaries, between 7 and 9 ovaries were analysed for each experimental group. Specific sample sizes are given in each relevant Materials and methods section and Figure legends.

### Immunohistochemistry

Immunohistochemistry (IHC) reactions were carried out on sections of cultured fetal or neonatal ovaries. Ovarian sections were rehydrated and antigen retrieval was performed in 0.01 M citrate buffer, (pH 6, Sigma Aldrich Ltd), followed by a blocking step with 20% normal goat serum (1x PBS, 5% bovine serum albumin) for 1 h at room temperature. Slides were incubated overnight at 4°C with primary antibodies in 20% goat serum (1× PBS, 5% BSA) in a humidified environment; this was followed by incubation with the appropriate secondary antibodies (Table 1). Primary antibody was omitted for negative controls. Slides were washed in PBS (Fisher Scientific UK Ltd) with 0.1% Triton X (PBSTx) between each step. For visualisation, counterstaining was done with 4,6-diamidino-2-phenylindole (DAPI; Invitrogen) at 1:5000 for 10 min to label cell nuclei. Slides were then mounted with Vectashield hard-set mounting medium (Vector Laboratories) and coverslipped. Photomicrographs were obtained using a Leica DM5500B microscope with a DFC360FX camera. Image analysis was carried out using ImageJ, with the assessor blind to treatment.

**Table 1:** Antibodies and conditions for immunohistochemistry.

Antibody	Manufacturer	Cat. no.	Species	Dilution	Secondary antibody		
					Antibody	Manufacturer	Cat. no.
BrdU	Abcam	6326	Rat	1:500	Goat anti-rat 568 nm	Invitrogen	A11077
CC3	Cell Signaling Technology	9661	Rabbit	1:500	Goat anti-rabbit 568 nm	Invitrogen	A11011
DDX4/MVH	Abcam	27591	Mouse	1:200	Goat anti-mouse 488 nm	Invitrogen	A21124
DDX4/MVH	Abcam	ab13840	Rabbit	1:200	Goat anti-rabbit 568 nm	Invitrogen	A11011
MDA	Abcam	6463	Rabbit	1:1000	Goat anti-rabbit 568 nm	Invitrogen	A11011
SYCP3	Abcam	97672	Mouse	1:200	Goat anti-mouse 488 nm	Invitrogen	A21124
γH2AX (phospho S139)	Abcam	ab22551	Mouse	1:200	Goat anti-mouse 488 nm	Invitrogen	A21124

Cat. no., catalogue number.

### IHC analysis

#### Synaptonemal complex protein 3

Assembly of the synaptonemal complex (SC) was examined using IHC with markers for synaptonemal complex protein 3 (SYCP3), allowing visualisations of the progression through meiotic prophase I. Every 6th ovary section was included in the analysis. Counts were made of all oocyte nuclei in leptotene, zygotene, pachytene or diplotene stage of meiotic prophase I (*n* = 6 for all groups, from two culture runs). Oocytes where the SC was seen to be assembling but had not yet fully formed, indicated by the presence of many thin short SYCP3 threads, were classified as pre-pachytene (leptotene/zygotene). Oocytes with fully synapsed SC, indicated by the presence of long and thick SYCP3 threads that were generally more spaced apart, were classified as pachytene. Finally, if the SC could be seen to be disassembling but still present, as indicated by the presence of short but thick fragments, oocytes were classified as being at the diplotene stage.

#### BrdU, DDX4, CC3 and γH2AX

To examine proliferation specifically in oogonia of fetal mouse ovaries, a double IHC was carried out for a marker of proliferation (BrdU) and for a germ cell-specific marker (DDX4); for analysis of proliferating and non-proliferating oogonia, DDX4+/BrdU+ and DDX4+/BrdU− cells were counted manually in paired ovaries: every 6th section was included in analyses (E12.5 ovaries: *n* = 6, from three culture runs, and in E13.5 ovaries: *n* = 6–7, from three culture runs). BrdU is an analogue of the nucleoside thymidine which can be incorporated in place of thymidine in newly synthesised DNA of proliferating cells. A double IHC was also carried out for γH2AX, a marker of double-strand DNA breaks (DSBs), and DDX4 in E12.5 ovaries in order to investigate levels of DNA damage within the oocytes. DDX4+/γH2AX+ and DDX4+/γH2AX− cells were counted manually from two non-consecutive sections in paired ovaries and the proportion of DDX4+/γH2AX+ cells was calculated as a percentage of total cells counted (*n* = 5, from two culture runs). Two non-consecutive sections from paired fetal E12.5 ovaries were also stained for the apoptotic marker cleaved caspase 3 (CC3). CC3 is an executioner caspase activated in an apoptotic cell by both the intrinsic (mitochondrial) and extrinsic (death ligand) apoptotic pathways. Analysis of CC3 expression was examined by measuring the area of signal as a percentage of ovary section area (E12.5 ovaries: *n* = 5 from three culture runs). From an initial image with merged fluorescent channels, the channels were split into a separate green (CC3) channel (Fig. 4E). From the split images, a threshold was set in order to analyse only cells positive for CC3 and to exclude any background signals or signals too small to be a cell.

To determine the level of both apoptosis and proliferation occurring in the neonatal ovary as a whole, a double IHC was carried out using antibodies to CC3 and to BrdU, with every 6th section included in the analysis (*n* = 8 for all groups, from three culture runs). Analysis of both CC3 expression and BrdU incorporation was examined by measuring the area of the signal as a percentage of the ovary section. From an initial image with merged fluorescent channels (Fig. 7Bi), the channels were split into separate green (CC3) or red (BrdU) channels. From the split images, a threshold was set in order to analyse only cells positive for either CC3 (Fig. 7Bii) or BrdU (Fig. 7Biii). By calculating the intensity values of the pixels, only in those areas selected in the grayscale image, the percentage area of CC3 expression and BrdU localisation was determined as a percentage of the total ovarian tissue (DAPI) section area.

#### MDA and DDX4

To examine the production of ROS following B[a]P treatment, a double IHC was performed utilising antibodies against a common marker of oxidative stress, malondialdehyde (MDA), alongside the germ cell marker DDX4. The area and intensity of signal as a percentage of ovary section area were analysed as described earlier. Three sections from each ovary were included, *n* = 9 from five culture runs.

### Statistical analysis

All statistical analyses were conducted using GraphPad prism (GraphPad Software, Inc.). Initially, data normality was assessed using Kolmogorov–Smirnoff tests and the variance of data sets was assessed by comparing the s.d.s of the control and B[a]P-treated ovaries. For experiments with more than two treatment groups, where there was normally distributed data, one-way ANOVA was performed, followed by the Bonferroni *post-hoc* test where ANOVA showed statistical significance between control and treatments. For data that was not normally distributed, Kruskal–Wallis non-parametric test was used, followed by Dunn’s *post hoc* test to determine the significance between the control and treatments. For the germ cell proliferation, DNA damage and apoptosis experiments in fetal E12.5 mouse ovaries, each B[a]P treated ovary was paired with a control ovary from the same fetus; therefore, paired two-tailed *t*-tests were used to analyse the two groups (control and B[a]P-exposed) for proliferation rate and germ cell number. For the germ cell proliferation study on E13.5 ovaries, meiotic progression study and the oxidative stress study, ovaries were pooled before being cultured in either control or B[a]P conditions; therefore, unpaired two-tailed *t*-tests were used. For the oxidative stress study, the variance was compared using an F test: an unpaired two-tailed *t*-test with Welch’s correction was used to compare the MDA expression of control and B[a]P-treated ovaries since there was a significant difference between the variance of the two groups. Results are given as mean ± s.e.m., with results considered statistically significant where *P* < 0.05.

## Results

### Exposure to B[a]P prior to follicle formation has a detrimental effect on formation of healthy follicles

**Figure 2:**
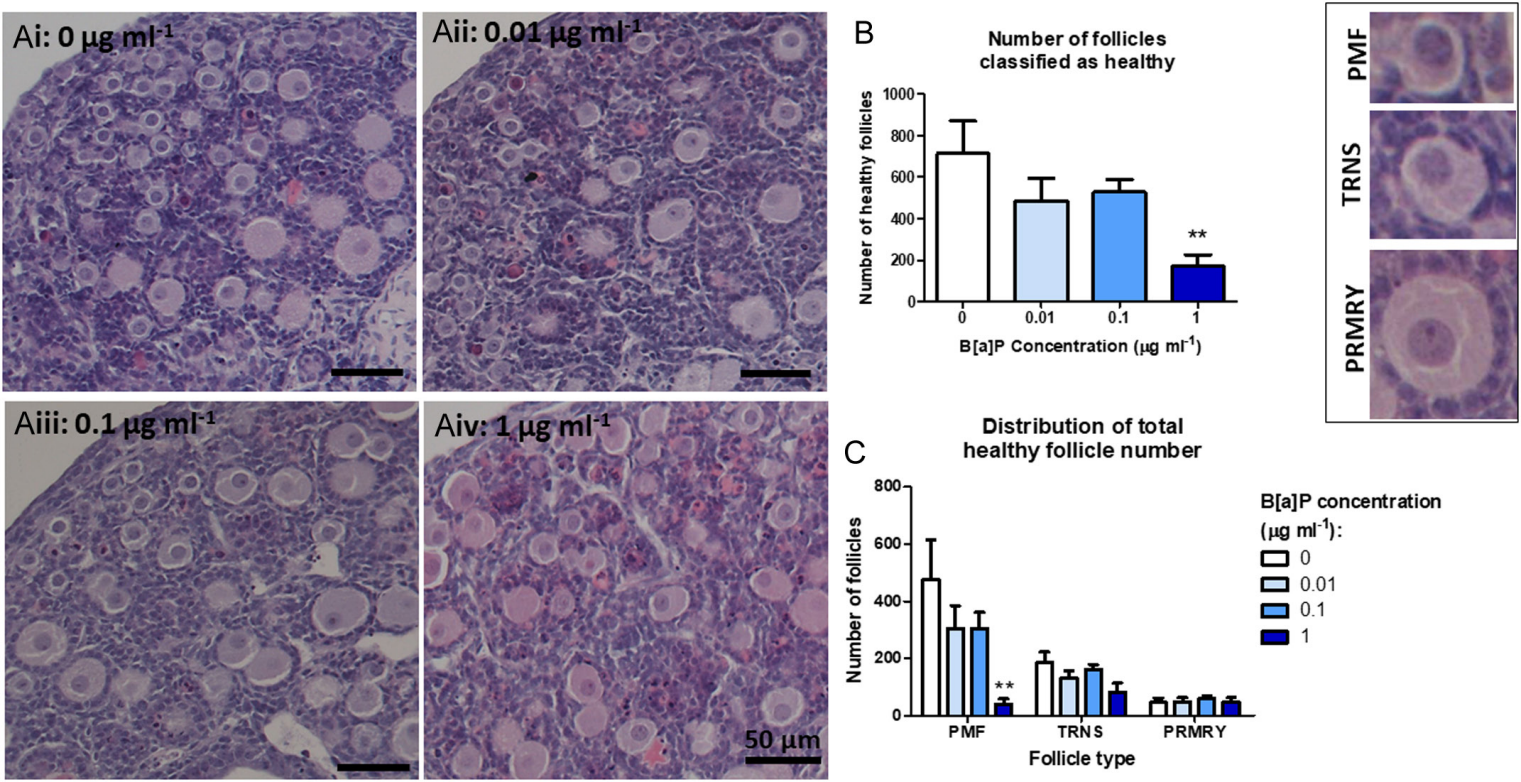
B[a]P affects the number and distribution of healthy follicles in the embryonic ovary. (Ai–Aiv) Photomicrographs of haematoxylin and eosin stained sections from embryonic mouse ovaries cultured with increasing concentration of B[a]P. Insert in the top right-hand corner shows representative images of PMF, TRNS and PRMRY follicles. Histograms show (B) total numbers and (C) distribution of the developmental stage of healthy follicles across different B[a]P exposure groups. Scale bars: 50 µm. Bars denote mean + s.e.m.; *n* = 7. Stars denote significant differences relative to control (**P < 0.01).

An initial dose–response experiment was carried out to examine the effects of B[a]P exposure on fetal ovary development from germ cell proliferation, entry into meiosis and germ cell nest breakdown up to follicle formation. Ovaries from E13.5 mouse embryos were cultured for 12 days and exposed to either 0.1% DMSO only (control) or a range of B[a]P concentrations (0.01, 0.1 or 1 µg/mL) for the first 6 days of culture (days 0–6), with day 6 of culture being equivalent to an E19.5 *in vivo* ovary. All ovaries were then cultured for a further 6 days in the control medium without DMSO and B[a]P (days 6–12), with day 12 of culture being at the equivalent stage as a PND4 *in vivo* ovary. Histological analysis was carried out on cultured ovaries and follicles were categorised by follicle stage and health (Fig. 2Ai-Aiv). B[a]P reduced the number of healthy follicles remaining in B[a]P-treated ovaries in a dose-dependent manner, reaching significance at the top dose of B[a]P (1 µg/mL), with numbers reduced to just 24% of controls (Fig. 2B. *P* < 0.01; *n* = 7). The surviving healthy follicles were further categorised to see which type of follicle was most affected (Fig. 2C), where the number of healthy PMFs was reduced by 92% following exposure to the top dose of B[a]P, when compared with controls (*P* < 0.01). No significant effect was found in the later stages of follicle development (TRNS *P* = 0.17; PRMRY *P* = 0.07). There was no difference in the total number of follicles classified as unhealthy across different treatment groups (Supplementary Fig. 1A, see section on supplementary materials given at the end of this article). However, the number of primordial and transitional follicles classified as unhealthy was reduced, whereas the number of primary follicles classified as unhealthy was increased (Supplementary Fig. 1B).

### B[a]P reduces the number of germ cells in the fetal mouse ovary but does not affect germ cell proliferation.

**Figure 3:**
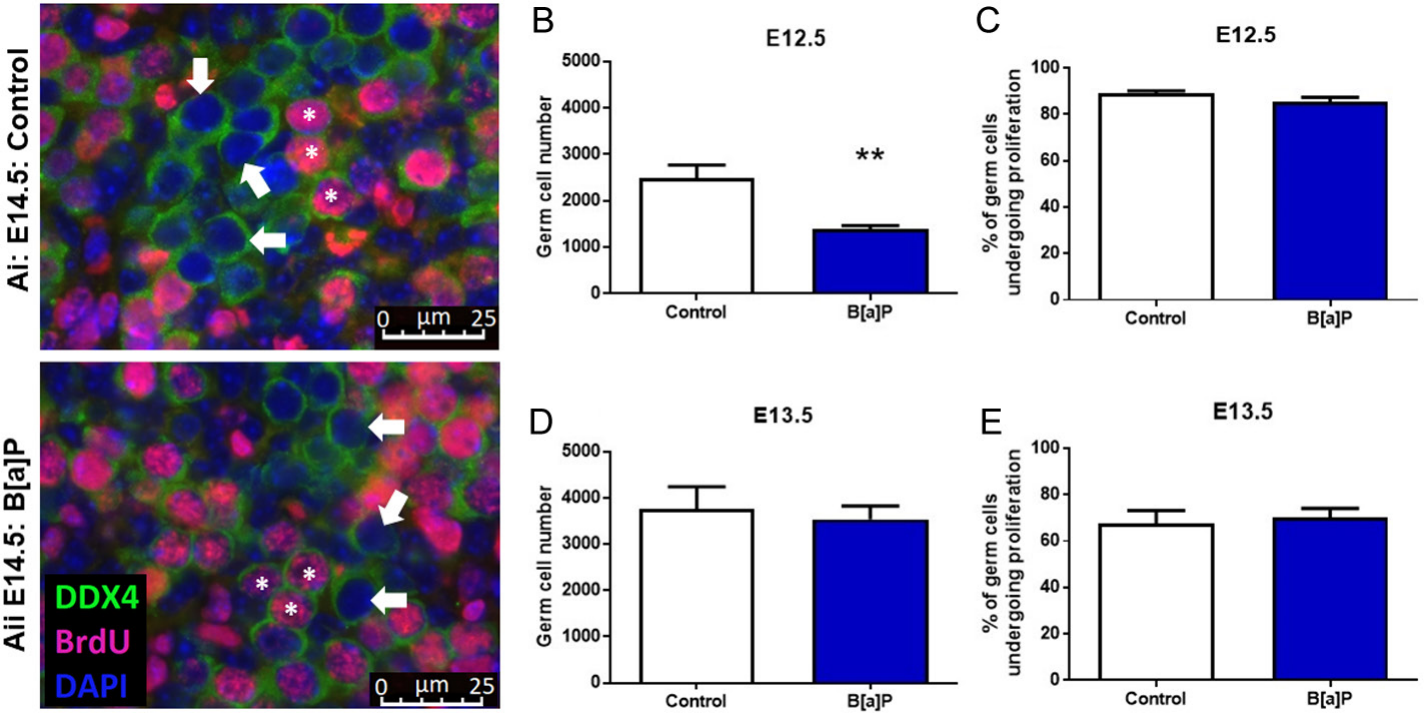
B[a]P reduces the number of germ cells in the fetal ovary but does not affect the proliferation of germ cells. (A) Representative photomicrographs of cultured E13.5 embryonic mouse ovaries cultured for 24 h under either (Ai) control conditions, or (Aii) with 1 µg/mL B[a]P, showing non-proliferating germ cells (DDX4-positive; green) and proliferating germ cells (DDX4-positive/BrdU-positive; green and pink). White arrowheads show examples of non-proliferating germ cells; asterisks show examples of proliferating germ cells. Histograms show total number of germ cells in ovaries cultured from (B) E12.5 ovaries and (D) E13.5 ovaries, as well as the percentage of germ cells undergoing proliferation during culture of (C) E12.5 ovaries and (E) E13.5 ovaries. Scale bars represent 25 µm. Bars denote mean + s.e.m.; *n* = 6 for both E12.5 and E13.5 treatment groups; *n* = 6 for the E13.5–E14.5 control group and *n* = 7 for the E13.5–E14.5 B[a]P-treated group. Stars denote significant differences relative to control (**P < 0.01).

Following the observed reduction in follicle numbers following B[a]P exposure prior to follicle formation, a further experiment was carried out to determine whether this reduction was due to an effect on germ cell proliferation. E12.5 or E13.5 mouse ovaries were cultured for 24 h in the presence of the high (1 µg/mL) dose of B[a]P or in control conditions. The number of germ cells that were either proliferating (Fig. 3A and B. DDX4+/Brdu+) or non-proliferating (Fig. 3A and B. DDX4+/BrdU−) was counted in both groups. A significant loss of germ cells was seen in B[a]P-exposed E12.5 cultured ovaries only (Fig. 3; *P* < 0.01, *n* = 6), with no effect on germ cell number when exposure occurred a day later, between E13.5 and E14.5 (3E; *P* = 0.702, *n* = 6). However, the observed reduction in germ cell number was not found to be due to an effect on the rate of germ cell proliferation: B[a]P had no effect on the rate of germ cell proliferation at either time point (Fig. 3D; E12.5 cultured ovaries *P* = 0.075, *n* = 6. Fig. 3F; E13.5 cultured ovaries *P* = 0.594, *n* = 6–7), with 85–90% of the germ cells proliferating in E12.5 cultured ovaries and 65–70% in E13.5 cultured ovaries in both the control and the treated groups.

### B[a]P increases DNA double-strand breaks in germ cells of the fetal mouse ovary but does not increase apoptosis levels.

**Figure 4:**
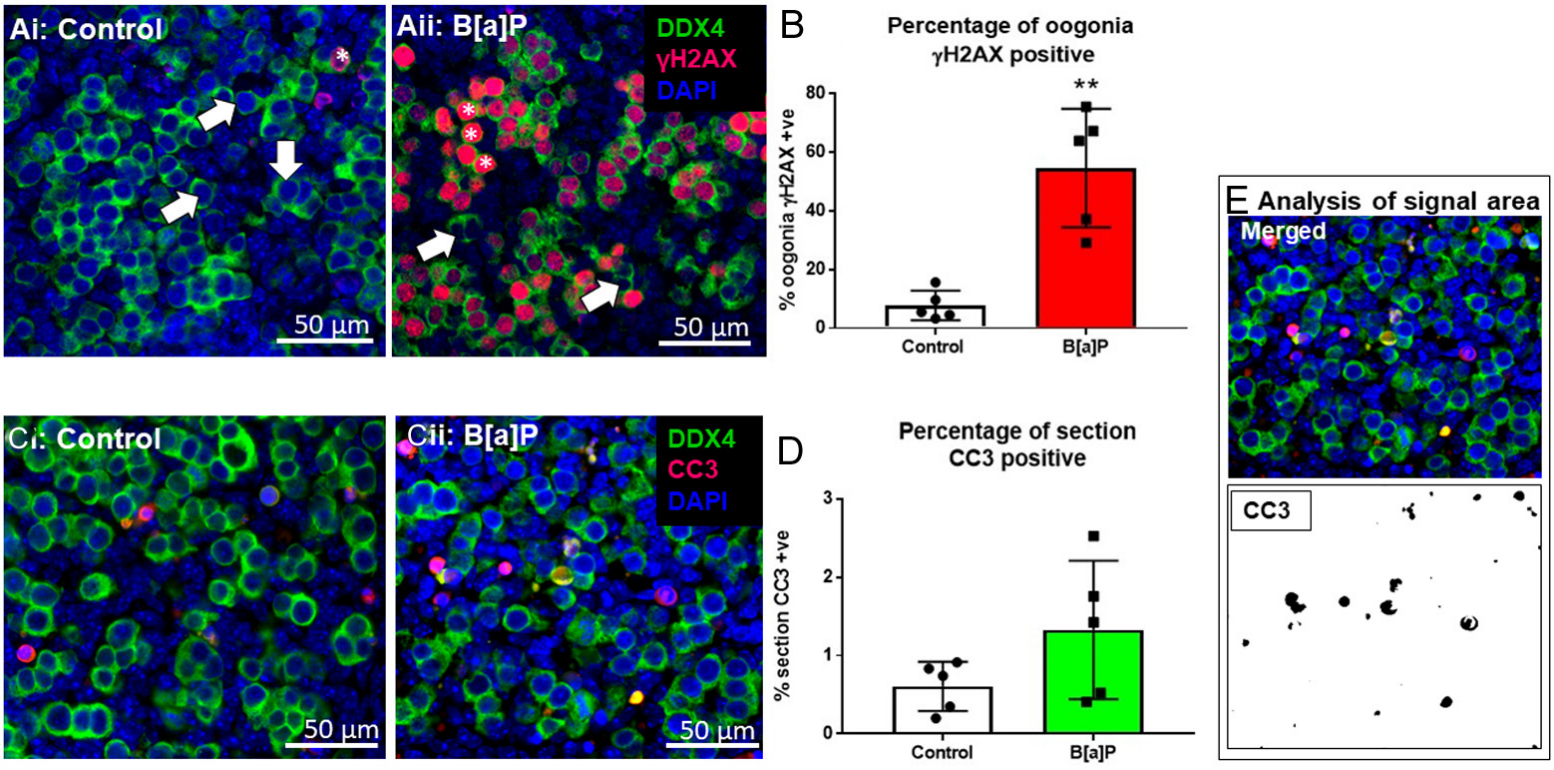
B[a]P increases DNA double-strand breaks within germ cells of the fetal ovary but does not significantly increase apoptosis levels. Representative images of E12.5 ovaries cultured for 24 h under either (Ai, Ci) control conditions or (Aii, Cii) with 1 µg/mL B[a]P. (Ai, Aii) Ovary sections show γH2AX positive oogonia (green, pink) and γH2AX negative oogonia (green). White arrowheads show examples of γH2AX negative oogonia; asterisks show examples of γH2AX positive oogonia. (B) Histogram shows the percentage of oogonia counted that were γH2AX positive. (Ci, Cii) Ovary sections show DDX4 localisation (green) and cells expressing CC3 (pink). (D) Histogram shows the percentage of tissue section area positive for CC3. To measure the area of CC3 expression. (E) The fluorophore area for CC3 was measured using ImageJ/Fiji software, as a percentage of the total area of the section. Scale bars represent 50 µm. Bars denote mean + s.e.m.; *n* = 5 for all. Stars denote significant differences relative to control (**P < 0.01).

Given that the number of germs cell decreased after the 24 h exposure of E12.5 ovaries to B[a]P, the levels of DNA damage and apoptosis were also examined in these ovaries. The number of germ cells with or without evidence of DNA damage (Fig. 4Ai,ii. DDX4+/γH2AX+ and DDX4+/γH2AX−, respectively) was counted in both groups. A significant increase in the proportion of γH2AX positive germ cells was seen after B[a]P exposure when compared with controls (Fig. 4B, *P* = 0.004, *n* = 5). In addition, E12.5 ovaries were also examined for expression of the apoptotic marker CC3 after a 24 h exposure to B[a]P or DMSO. There was an observed trend towards an increase in the percentage area of CC3 expression per total tissue section area, but this increase was not statistically significant (Fig. 4D, *P* = 0.119, *n* = 5)

### B[a]P decreases the proportion of oocytes at pachytene and diplotene stages of prophase I.

**Figure 5:**
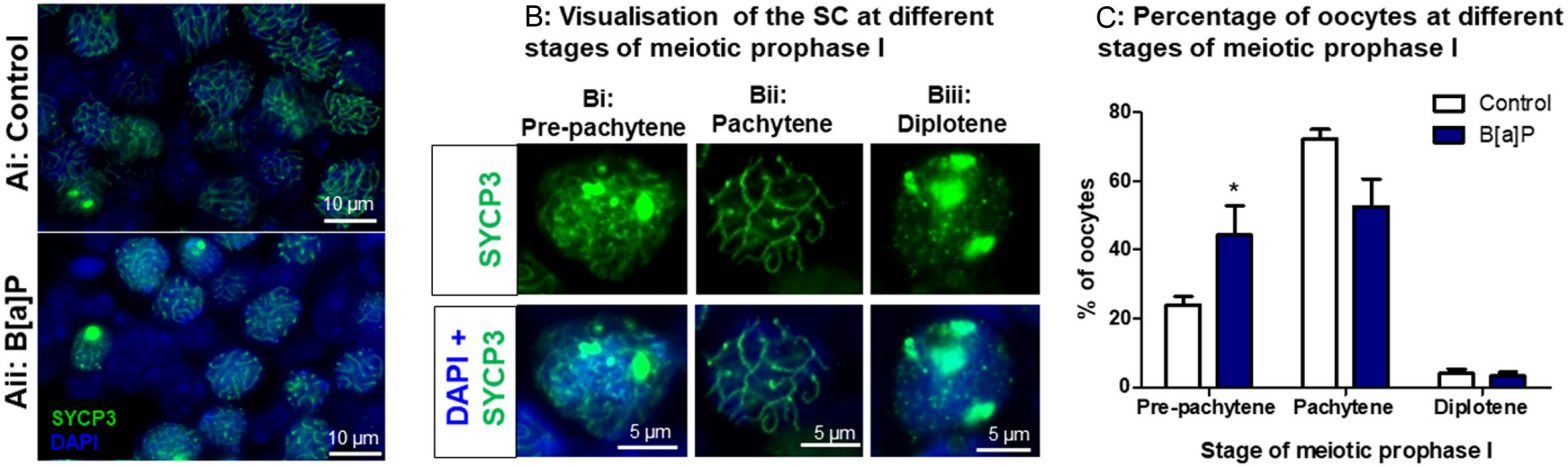
B[a]P increases the proportion of oocytes at pre-pachytene stages of prophase I. (A–B) Representative images of E13.5 ovaries cultured for 72 h under either (Ai) control conditions or (Aii) with 1 µg/mL B[a]P and stained for SYCP3 (green) in order to assess the synaptonemal complex (SC). Oocytes were categorised into different stages of prophase I depending on the stage of SC formation. Oocytes were classed as pre-pachytene (leptotene or zygotene) when the SC was seen to be assembling (Bi), whereas oocytes with fully synapsed SC, were categorised as pachytene stage oocytes (Bii). Finally, if the SC could be seen to be disassembling, but still present, oocytes were classed as being at the diplotene stage (Biii). Histogram shows the percentage of oocytes at pre-pachytene (leptotene/zygotene), pachytene or diplotene stages in control vs B[a]P-treated ovaries (C). Scale bars represent 10 µm in (A) and 5 µm in B(i) and (ii). Bars denote mean + s.e.m.; *n* = 6. Star denotes significant differences relative to control (*P < 0.05).

The effect of B[a]P on meiosis I was examined in fetal ovaries. E13.5 mouse ovaries that had been cultured for 72 h either in the presence of 1 µg/mL B[a]P or in a control medium were immunostained with antibodies for SYCP3 (Fig. 5A and B), a critical component of the SC. As expected, around 24% of oocytes in control ovaries were at pre-pachytene stages (leptotene or zygotene), 72% at the pachytene stage and 4% at the diplotene stage, after 72 h of culture (Stefansdottir *et al.* 2016). However, the proportion of oocytes at pre-pachytene stages had increased to 44% in B[a]P-exposed ovaries (Fig. 5Ci; *P* = 0.047, *n* = 6). Consequently, a smaller proportion, or 53%, of pachytene-stage oocytes were observed in the B[a]P-treated ovaries, although this was not significantly different from control ovaries (Fig. 5Cii, *P* = 0.093, *n* = 6). There was no effect on the proportion of diplotene-stage oocytes in the treated vs control ovaries (Fig. 5Ciii, *P* = 0.704, *n* = 6).

### B[a]P impacts the number and health of follicles in mouse ovaries exposed after follicle formation.

**Figure 6:**
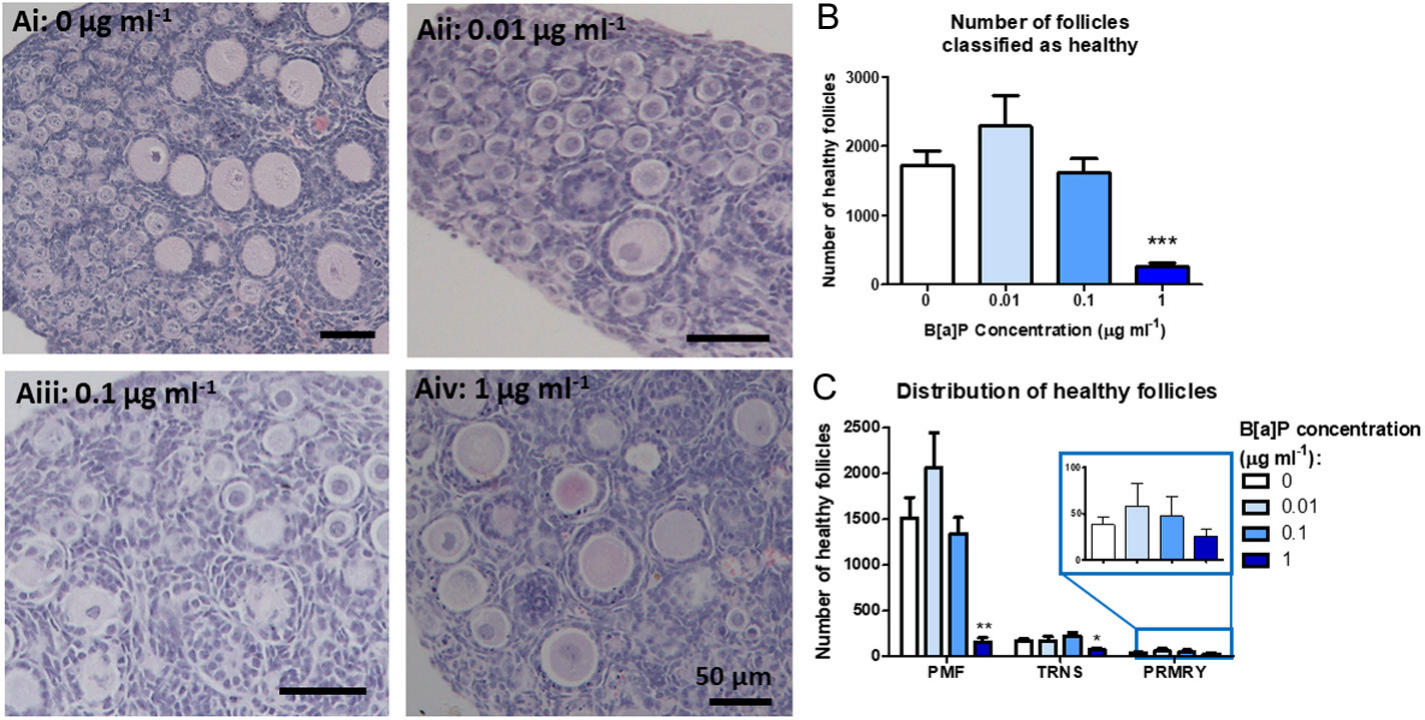
Neonatal ovaries cultured with B[a]P contain fewer healthy follicles. (A) Representative histological sections of neonatal ovaries cultured for 6 days in either (Ai) control conditions, or with increasing concentrations of B[a]P: (Aii) 0.01 µg/mL, (Aiii) 0.1 µg/mL or (Aiv) 1 µg/mL. Histograms show (B) the number and (C) the distribution of healthy follicles across different B[a]P treatment groups. Scale bars: 50 µm. Bars denote mean + s.e.m.; *n* = 8 for control group, *n* = 7 for 0.01 µg/mL 10 and 0. 1 µg/mL group and *n* = 9 for 1 µg/mL group. Stars denote significant differences relative to control (*P < 0.01, **P < 0.01, ***P < 0.001).

In order to examine the effect of B[a]P on follicles after follicle formation, neonatal (PND4) ovaries were cultured for 6 days in either a control medium or exposed to increasing doses of B[a]P for the duration of the culture (*n* = 8 for control, 7 for the 0.01 and 0.1 µg/mL groups and 9 for the 1 µg/mL group). Follicle number and health were assessed in histological sections of cultured ovaries (Fig. 6Ai-Aiv). Ovaries treated with 1 µg/mL B[a]P had 85% fewer healthy follicles than control ovaries (Fig. 6B; *P* < 0.001), with no effect found at lower doses of B[a]P. When the healthy follicles were further analysed by follicle stage, it was evident that the drastic drop in the number of healthy follicles was primarily due to a significant decrease in the number of healthy PMFs (Fig. 6C; mean ± s.e.m. number of healthy PMFs in 1 µg/mL B[a]P group 158 ± 47, vs control 1513 ± 224, *P* < 0.001) and to a lesser extent TRNS follicles (mean ± s.e.m., number of healthy TRNS in 1 µg/mL B[a]P group 71 ± 14, vs control 165 ± 24, *P* = 0.0058) with no effect was observed on the number of healthy PRMRY follicles (Fig. 6C; *P* = 0.481). The number and distribution of unhealthy follicles were also examined, where the number of follicles classified as unhealthy significantly increased in the 1 µg/mLgroup, with a drastic increase in unhealthy PMFs (Supplementary Fig. 1C and D).

### B[a]P increases apoptosis and reduces cell proliferation in ovarian follicles

**Figure 7:**
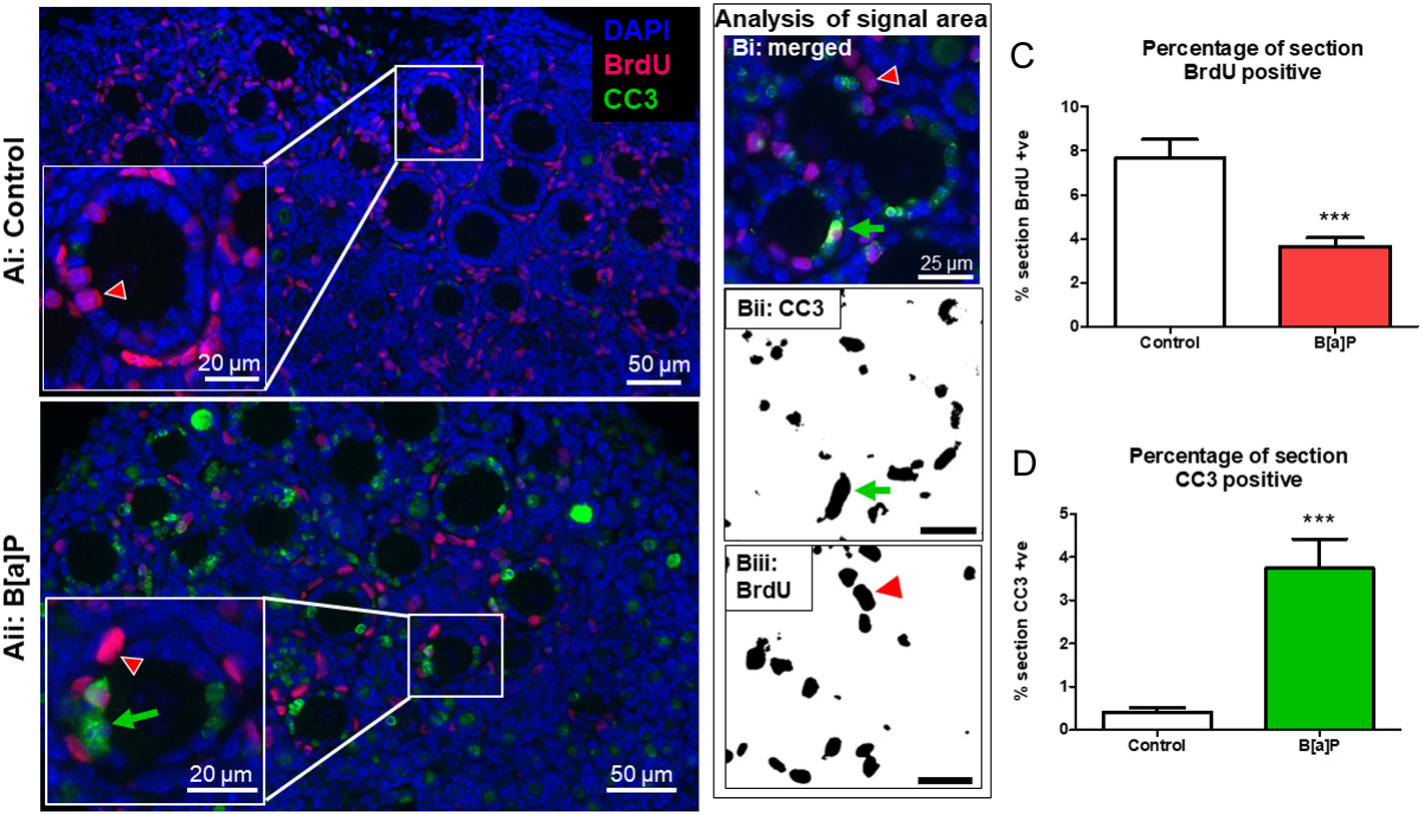
Apoptosis is increased, while somatic cell proliferation is decreased, following exposure to B[a]P. (A) Representative photomicrographs of cultured neonatal (Ai) control and (Aii) B[a]P treated tissues showing the localisation of CC3 (green) and BrdU (red), counterstained with DAPI (blue). Insets show higher magnification images of ovarian follicles. (B) Each fluorophore area (except for DAPI) was measured as a percentage of total area of the section using ImageJ/Fijij software. (C) Histograms show the percentage of the tissue section that is BrdU positive and (D) the percentage of tissue section that is positive for CC3. Green arrows show examples of cells expressing CC3 and red arrowheads show the localisation of BrdU within cells. Scale bars denote 50 µm, 20 µm in insets (Ai, ii) or 25 µm for images showing analysis of signal area (Bi-iii). Bars denote mean + s.e.m.; *n* = 8 for both groups. Stars denote significant differences relative to control (***P < 0.001).

The dramatic loss in follicle number and health following B[a]P exposure in cultured PND4 ovaries prompted an examination of whether this was due to B[a]P inducing apoptotic cell death and/or changes in the proliferative ability. PND4 mouse ovaries were cultured in either control conditions or with the high (1 µg/mL) dose of B[a]P and examined for a marker of apoptotic cell death (CC3) or cell proliferation via the incorporation of BrdU into ovarian cells (Fig. 7Ai–ii and Bi–iii). There was a significant reduction in the rate of BrdU incorporation into somatic cells following B[a]P exposure (Fig. 7C; area of BrdU incorporation mean % + s.e.m.: B[a]P group: 3.65 ± 0.4, vs control group: 7.66 ± 1.0, *P* < 0.001, *n* = 8 for both treatment groups). Alongside that effect, there was also an increased proportion of tissue expressing the apoptotic marker CC3 in response to B[a]P exposure (Fig. 7D; 0.4 ± 0.1% of the area of control ovaries was positive for CC3, whereas in B[a]P-treated ovaries, this had risen to 3.7 ± 0.7%, *P* < 0.001, *n* = 8 for both treatment groups).

### Expression of an oxidative stress marker, MDA, in response to B[a]P exposure

**Figure 8:**
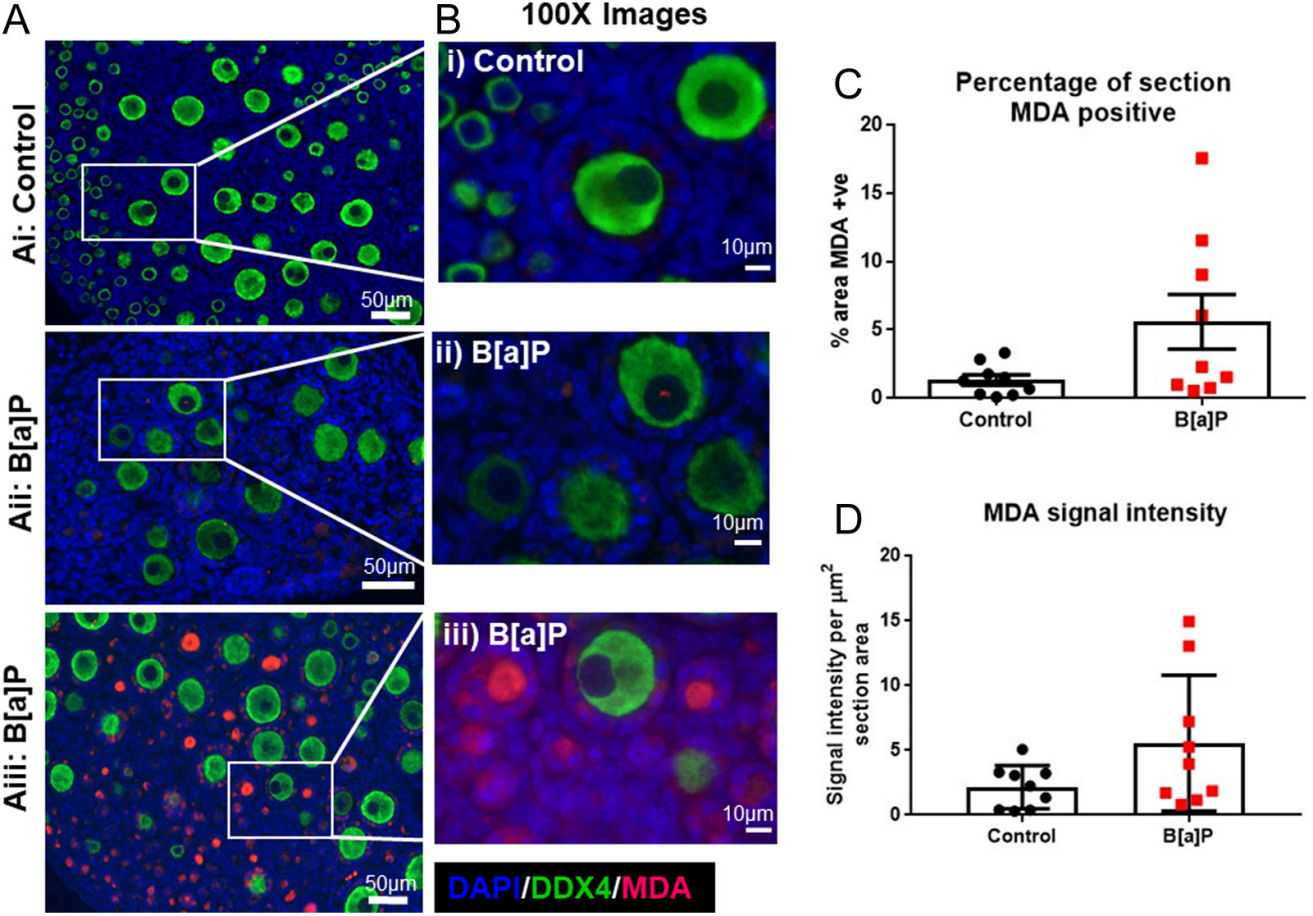
B[a]P does not significantly affect ovarian MDA expression. (A) Representative photomicrographs of (Ai) control and (Aii, Aiii) B[a]P-treated cultured ovaries showing immunohistochemical localisation of MDA (red), DDX4 (green) and DAPI (blue). Due to the large variation of MDA signalling observed in B[a]P ovaries, representative images from ovaries with low (Aii, Bii) and high (Aiii, Biii) levels of MDA expression have been shown. (B) Shows higher power (100×) magnification images of (Bi) control and (Bii, Biii) B[a]P-treated ovaries. (C) Histogram shows the percentage of tissue area positive for MDA for both groups. (D) Histogram shows the intensity of MDA expression per tissue area for both groups. Scale bars denote 50 µm, or 10 µm in the high-power images. Bars denote mean + s.e.m.; *n* = 9 for both groups.

In order to gain further insight into the mechanisms behind B[a]P-induced follicle loss, neonatal (PND4) mouse ovaries were cultured for 6 days either in a control medium or exposed to the high (1 µg/mL) concentration of B[a]P. Ovaries were fixed and immunostained for a marker of oxidative stress, MDA, an aldehyde end-product of ROS-induced damage (Fig. 8A and B). MDA is an aldehyde end-product of ROS-induced damage to the polyunsaturated fatty acids of phospholipid membranes, a process known as lipid peroxidation. There was a trend towards an increased proportion of area positive for MDA in B[a]P-treated ovaries (5.6 ± 2.0%) when compared with controls (1.3 ± 0.4%); however, this was not significant (Fig. 8C; *P* = 0.068, *n* = 9 for both groups). In addition, when examining the intensity of MDA expression per section area (µm^2^), a similar trend was observed, where increased intensity in B[a]P-treated ovaries (5.6 ± 1.8 per µm^2^) was observed when compared with control ovaries (2.2 ± 0.6 per µm^2^), but again, this did not reach significance (Fig. 8D; *P* = 0.095, *n* = 9 for both groups). For both, the proportion of the area positive for MDA (*P* < 0.001) and the intensity of MDA expression (*P* < 0.01), there was a significant difference in the variance of the control and B[a]P-treated groups.

### Melatonin does not ameliorate the damaging effects of B[a]P on the neonatal mouse ovary *in vitro*

**Figure 9:**
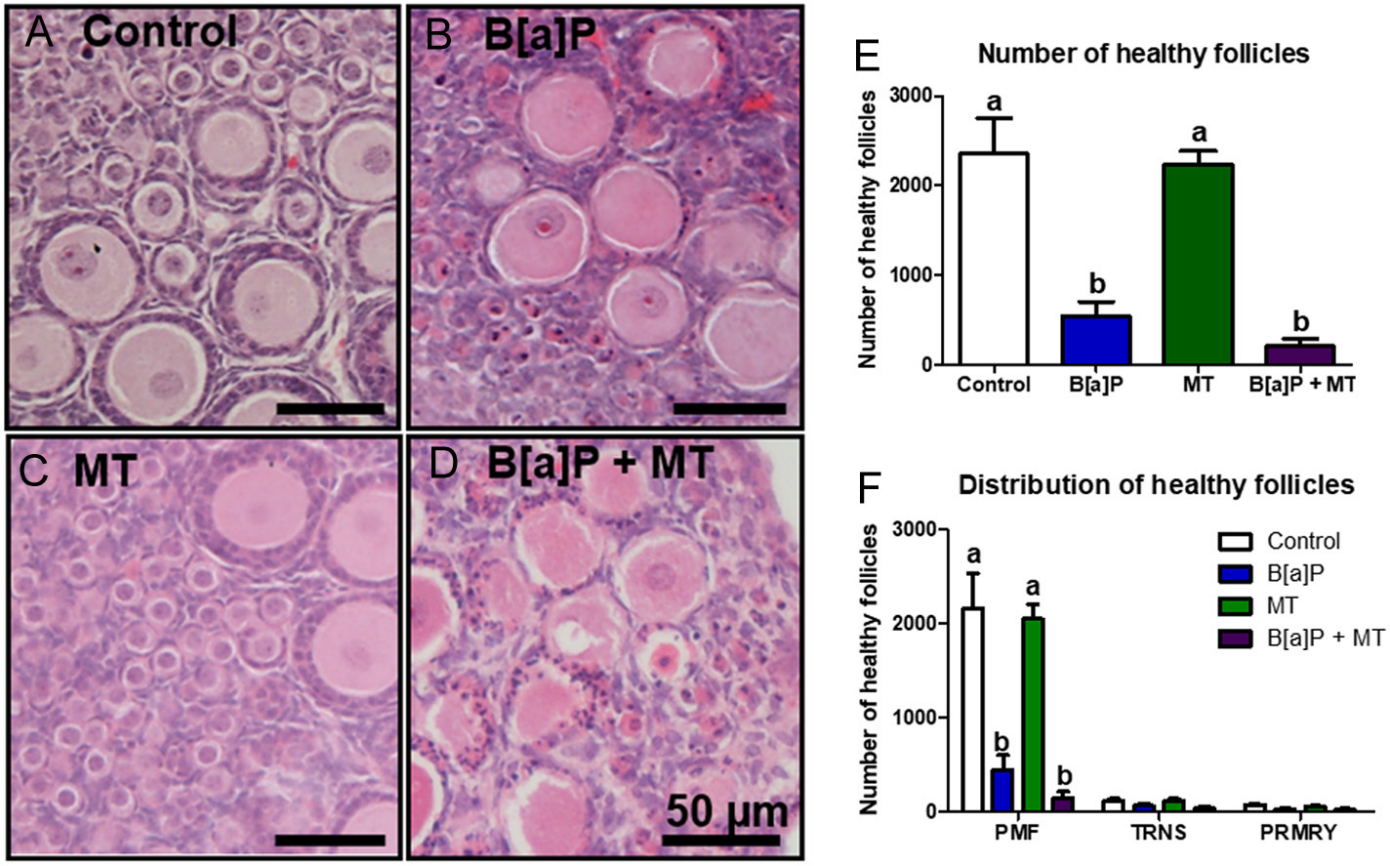
Melatonin does not protect the ovary from B[a]P-induced depletion of healthy follicles. (A–D) Representative photomicrographs of (A) control, (B) B[a]P-treated, (C) MT-treated and (D) B[a]P + MT-treated ovaries, stained with haematoxylin and eosin. Histograms show the (E) total number of healthy follicles and (F) distribution of healthy follicles in ovaries following exposure to B[a]P alone (blue), MT alone (green) or both B[a]P and MT (purple). Bars denote mean + s.e.m.; *n* = 6 for all experimental conditions. Columns with different letters are statistically significant to each other (*P* < 0.05).

Given that there was a trend towards increased oxidative stress following B[a]P exposure, a further experiment was carried out to determine whether the antioxidant melatonin could protect ovarian follicles from the observed B[a]P-induced follicle loss: neonatal (PND4) ovaries were cultured for 6 days in either control conditions, B[a]P (1 µg/mL) only, melatonin (10 µg/mL) only or both B[a]P and melatonin. Histological analysis showed that there was no evidence to suggest that the administration of melatonin ameliorates the follicle health of B[a]P-treated ovaries (Fig. 9). Treatment with melatonin alone had no adverse effect on the ovary, with no significant differences found in the number of healthy or unhealthy follicles between the control and melatonin-only group, at all stages of follicles analysed (Fig. 9E and F). Both the B[a]P group and the B[a]P + melatonin groups contained significantly fewer healthy follicles than the control group and the melatonin-only group (Fig. 9E; % healthy follicles mean ± s.e.m.: control group: 97.8 ± 0.8%, B[a]P group: 64.4 ± 11.7%, MT group: 94.9 ± 1.9%, B[a]P + MT group: 43.2 ± 16.2%, *P* < 0.05). Furthermore, no difference was found in the number of healthy follicles in ovaries exposed to B[a]P alone when compared with the B[a]P + melatonin group (Fig. 9E; *P* > 0.05). Similarly, when healthy follicles were analysed by follicle type, there was no evidence for melatonin ameliorating the previously observed B[a]P-induced loss of healthy PMFs (Fig. 9F).

## Discussion

The PAH B[a]P is a ubiquitous environmental pollutant and carcinogen that is found not only in the tar of cigarette smoke but also in grilled food, coal and roof tar, contaminated water, petroleum products and polluted air or haze. Haze comes from industrial pollution, motor vehicle exhaust, dust and coal combustion, when organic matter and fossil fuels undergo incomplete combustion, releasing PAHs such as B[a]P into the atmosphere (Omar *et al.* 2006, Ramakreshnan *et al.* 2018, Sulong *et al.* 2019). B[a]P is a potent ovotoxicant that can disrupt ovarian function, leading to reduced fertility and lower conception rates in IVF (Neal *et al.* 2007, Neal *et al.* 2008, Sadeu & Foster 2011, Archibong *et al.* 2012, Sobinoff *et al.* 2012). It has also been linked to premature ovarian insufficiency through early depletion of the PMF pool (Mattison & Thorgeirsson 1979, Kummer *et al.* 2013, Lim *et al.* 2015). Given its ubiquitous nature, it is important to improve our understanding of the reproductive impact of B[a]P on the developing fetus, including the ovary which is particularly vulnerable to xenobiotic exposure during fetal development (Kristensen *et al.* 1995, Ernst *et al.* 2012, Anderson *et al.* 2014, Hoyer & Keating 2014, Camlin *et al.* 2016, Lea *et al.* 2016, Lecante *et al.* 2021).

To date, numerous studies have investigated the damaging effect of PAHs including B[a]P on the rodent ovary, with the majority of this research focussing on effects on later stages of ovarian development, examining pre-antral and antral follicles, oocyte maturation and fertilisation (Neal *et al.* 2007, Archibong *et al.* 2012, Sobinoff *et al.* 2012, Sadeu & Foster 2013, Sobinoff *et al.* 2013, Zhang *et al.* 2018). Through these studies, it has been shown that B[a]P exposure can induce follicle loss due to both accelerated PMF activation (Sobinoff *et al.* 2012) and increased apoptosis (Sobinoff *et al.* 2013, Rahmani *et al.* 2021). There is also evidence to suggest that B[a]P may induce cytotoxicity in ovarian cells through increased production of ROS (Sobinoff *et al.* 2012, Miao *et al.* 2018, Zhang *et al.* 2018, Rahmani *et al.* 2021). An imbalance between the production and scavenging of ROS, which leads to an excessive level of ROS, can result in the decline of the mitochondrial membrane potential in cells: this can promote carcinogenic and mutagenic potential, resulting in DNA damage within cells and ultimately, apoptosis (Tamura *et al.* 2012, Archibong *et al.* 2018). Furthermore, B[a]P can compromise meiotic progression of maturing oocytes resulting in oocyte meiotic failure and in the generation of aneuploid eggs (Zhang *et al.* 2018). However, less is known about the effect of B[a]P on the developing fetal ovary, around the time of follicle formation. Prenatal exposure to maternal cigarette smoke has been shown to significantly reduce the number of germ and somatic cells in the developing human fetal ovary (Lutterodt *et al.* 2009, Mamsen *et al.* 2010). However, in part, given the difficulty in studying the early stages of germ cell development due to the inaccessibility of human fetal ovarian tissue, the molecular mechanisms underlying the adverse effect of B[a]P and other PAHs on the development of the ovary have been limited (Ge *et al.* 2012). As a result, it is uncertain whether B[a]P exposure affects germ cell proliferation, the ability of oogonia to enter and progress through prophase I of meiosis or their ability to then form healthy follicles. Here, we have examined the effect of B[a]P on the developing mouse ovary *in vitro* where we have shown that B[a]P can damage the ovary, both before and shortly after follicles have formed.

### Fetal concentrations of B[a]P

The concentrations of B[a]P used in this study (up-to 1 µg/mL) are comparable, or lower, than those previously reported in other studies, which were up to 1 µg/mL, up to 10 µg/mL and 60–70 µg/mL,respectively (Tuttle *et al.* 2009, Lim *et al.* 2016, Miao *et al.* 2018). It is, though, possible that the highest dose of B[a]P used here is higher than the concentration that a human fetus is likely to be exposed to (Neal *et al.* 2008, Fowler *et al.* 2014, Machado *et al.* 2015). However, given B[a]P’s ubiquitous nature, it is difficult to estimate exactly how much B[a]P a pregnant woman, and consequently her fetus, could be exposed to. The level of maternal exposure is likely to vary considerably based on a variety of factors, including whether they smoke or not, the amount of chargrilled meat they consume, whether they have any occupational exposure to PAHs, and where they live, with exposure likely higher in cities than in rural areas, and bearing in mind that increased haze has become a large-scale pollution problem in many in southeast Asian nations (Ramakreshnan *et al.* 2018). It has been estimated that each cigarette smoked contains between 3.36 and 200 ng of B[a]P, with the levels of B[a]P in female smokers’ serum and follicular fluid measuring around 0.98 and 1.32 ng/mL, respectively (ATSDR 1990, Kaiserman & Rickert 1992, Lodovici 2004, Neal *et al.* 2008). In addition, the amount of B[a]P taken in through the diet has been suggested to range between 8.4 and 17 µg/per person/day (Ramesh *et al.* 2010). PAHs can cross the placenta and enter the fetal bloodstream, with a near 3-fold increase in B[a]P concentrations reported in the umbilical cord of pregnant smokers when compared to non-smoking controls (1.13 vs 0.39 µg/mL, respectively) (Machado *et al.* 2015). The concentration of B[a]P in the liver of smoke-exposed fetuses has been measured at 1.37 µg/kg, compared to below detection levels in non-smoking pregnancies (Fowler *et al.* 2014). Worryingly, levels found in umbilical cord blood have been reported to generally exceed those found in paired maternal blood (Barr *et al.* 2007, Karttunen *et al.* 2010, Sexton *et al.* 2011).

### Follicle number and health

To investigate the potential impact of B[a]P exposure on the developing fetal ovary prior to and following the formation of the PMF, mouse ovary cultures were carried out that spanned either the period leading up to PMF formation, or the period immediately following PMF formation. Exposure to B[a]P prior to follicle formation, around the time of their entry into prophase I of meiosis, led to a reduction in germ cell number, with a substantial reduction in the number of healthy follicles subsequently formed. Even when B[a]P exposure occurred after the follicles had already formed, when the follicular environment could potentially have conferred some protection on the germ cells, the same concentration of B[a]P also resulted in a drastic reduction in follicle health and number. These findings support previous *in vivo* work, where gestational exposure to either B[a]P or cigarette smoke has been shown to deplete germ cells within the mouse and human fetal ovary, resulting in a reduction in fecundability (MacKenzie & Angevine 1981, Vähäkangas *et al.* 1985, Weinberg *et al.* 1989, Lutterodt *et al.* 2009, Mamsen *et al.* 2010). Furthermore, the work is also in agreement with previous *in vitro* work where B[a]P exposure to the E13.5 mouse fetal ovary for 48 h in culture resulted in a similar effect on germ cell depletion (Lim *et al.* 2016). Results here add to those of Lim *et al.* (2016) by focusing specifically on the effects of B[a]P during the early stage of ovary development in which germ cells are still undergoing proliferation, then entering into prophase I of meiosis.

### Effects of B[a]P on germ cell proliferation and health in the developing ovary

PAHs such as B[a]P can exert their toxic effect on the ovary through binding to the AhR, which is expressed in both mouse and human oocytes (Robles *et al.* 2000, Anderson *et al.* 2014). There is evidence that activation of AhR results in a reduction of germ cell proliferation in the human fetal ovary (Anderson *et al.* 2014). This effect was not seen in the mouse, with results by Lim *et al.* (2016) showing no impact of B[a]P on germ cell proliferation rates (Lim *et al.* 2016). However, Lim *et al.* (2016) examined proliferation relatively late in fetal ovary development, when most germ cells entered meiosis (McLaren & Southee 1997). Here, we looked at earlier time points when germ cell proliferation rates are still high, with the results providing further evidence to support the conclusion that B[a]P has no significant effect on germ cell proliferation in the developing mouse ovary. Given that there was a reduction in germ cell number in the B[a]P-exposed E12.5 ovaries, but that there was no evidence that this reduction was due to a decrease in the rate of germ cell proliferation, we subsequently examined the health of the oogonia by measuring levels of apoptosis and DNA damage. There was a marked increase in the amount of DNA DSBs present within B[a]P-exposed oogonia. However, while a similar trend was observed for apoptosis, this was not significant. While it is possible that the oogonia have been lost via an alternative cell death pathway, it may well be that by the time point examined the peak of CC3 expression has already been reached and that by the time oogonia are lost, CC3 expression has begun to fall. Regardless, these results show evidence of widespread DNA damage within the B[a]P-exposed oogonia: if left unrepaired, it could potentially have subsequent effects on any future offspring, even resulting in transgenerational effects.

### Prophase I of meiosis

Previous work on mouse and porcine oocytes have shown that B[a]P impacts later stages of meiotic progression, with B[a]P affecting the meiotic apparatus, disrupting normal spindle assembly and chromosome alignment, consequently leading to a meiotic failure (Miao *et al.* 2018, Zhang *et al.* 2018, Sui *et al.* 2020). However, these studies examined oocytes already in meiotic arrest, or during later stages of meiotic progression following ovulation and fertilisation of the oocyte. Progression through prophase I of meiosis is crucial for the development of the oocyte, during which time the SC forms between homologous chromosomes: when meiosis I is perturbed, oocyte loss can occur (Zickler & Kleckner 2015). Here, we report a significant increase in the proportion of germ cells still at pre-pachytene stages of prophase I 72 h after exposure to B[a]P. There are several possible explanations for this. The effect could be due to a delay in entry or progression through meiosis I in B[a]P-exposed oocytes. Germ cells may be lost as they reach the pachytene stage, resulting in the presence of proportionally more pre-pachytene stage oocytes. Alternatively, given that there were increased levels of DSBs 24 h after B[a]P exposure, and bearing in mind that DSBs do occur during meiotic prophase in order to initiate homologous recombination, an alterntuve explanation is that B[a]P could have accelerated primordial germ cell (PGC) entry into meiosis, although it is important to note that this would need to be followed by either a slowing down in the rate of meiosis, or loss of these early entry oocytes from the pachytene stage onwards. Of note, a previous study examining another PAH and AhR ligand, 9, 10-dimethylbenz[a]anthracene, on chicken germ cell development found a similar effect, with a reduction in the ability of PGCs to initiate meiosis (Ge *et al.* 2012). To the best of our knowledge, this is the first report of an effect of B[a]P on the ability of mammalian oocytes to progress through the early stages of prophase I of the first meiotic division.

### Ovarian health

There are some inconsistencies in the literature regarding whether the loss of ovarian follicles following exposure to B[a]P is due to increased activation of PMFs (Tuttle *et al.* 2009, Sobinoff *et al.* 2012) or to increased apoptosis (Lim *et al.* 2016, Miao *et al.* 2018). In the current study, there was a significant increase in apoptosis in ovaries that had been exposed to B[a]P after follicle formation. Expression of CC3 was observed in both follicular and interstitial tissue: it is not possible to definitively identify CC3-positive oocytes since oocyte expression of DDX4 decreases when the cell starts to undergo apoptosis (unpublished observation). The increase in apoptosis also corresponded with a reduced rate of somatic cell proliferation. Together, these findings are in support of previous work reporting follicle loss via apoptotic pathways following exposure to either B[a]P (Lim *et al.* 2016), or other PAHs also found in cigarette smoke (Matikainen *et al.* 2002). Not all studies have reported evidence of apoptosis in ovaries following exposure to B[a]P even at higher concentrations than those used in the current study. This could be due to alternative cell death pathways being involved in follicle depletion and/or due to these studies having missed the window of expression of apoptotic markers (Tuttle *et al.* 2009). Furthermore,Sobinoff *et al.* (2012), finding an increased proportion of growing follicles in mice exposed to B[a]P, proposed that B[a]P exposure leads to an acceleration of PMF activation (Sobinoff *et al.* 2012). Results here show no evidence of acceleration in PMF activation, with follicle loss primarily due to a drastic reduction in PMF numbers, and with no effect on the number of PRMRY follicles. This result is in line with several other studies showing that B[a]P exposure dramatically impacts on the number of PMFs (Mattison & Thorgeirsson 1979, Borman *et al.* 2000, Tuttle *et al.* 2009, Lim *et al.* 2015). Furthermore, the results presented bySobinoff *et al.* (2012) could also be explained solely by a dramatic reduction in PMF numbers, thus leading to the survival of proportionally more growing follicles.

Given that B[a]P exposure to ovarian follicles shortly after their formation resulted in a rise in apoptotic cell death, subsequent work explored whether this was due to an increase in ROS and oxidative stress, examining the expression of MDA. Perhaps surprisingly, there was no significant effect on MDA expression following B[a]P exposure. There was considerable variation in the level of MDA expression following exposure to B[a]P with significantly greater variance in the B[a]P-treated group than in control ovaries. There have been several studies reporting B[a]P-induced damage to the rodent and porcine ovary through increased levels of mitochondrial ROS and lipid peroxidation (Sobinoff *et al.* 2012, Miao *et al.* 2018, Zhang *et al.* 2018, Rahmani *et al.* 2021). In addition, studies exposing the fetal or neonatal rodent ovary to cigarette smoke, have also reported elevated levels of oxidative stress markers (Sobinoff *et al.* 2012, Camlin *et al.* 2016).

Miao *et al.* (2018) reported that the antioxidant melatonin can protect porcine oocytes from B[a]P-induced rise in oxidative stress, ensuring normal follicular development (Miao *et al.* 2018). Melatonin is a free radical scavenger that reduces oxidative stress during oocyte maturation and embryo development (Tamura *et al.* 2008, Tamura *et al.* 2012, Voiculescu *et al.* 2014). Given that there was an observed trend towards an increased ROS level following B[a]P exposure here, although this was insignificant, subsequent work investigated whether melatonin could ameliorate the ovarian damage caused by B[a]P; there was no evidence of a protective effect of melatonin on either follicle number or health following B[a]P exposure. The inconsistency between the finding reported here and the study by Miao *et al.* (2018) could be due to several factors. Miao *et al.* (2018) investigated cumulus–oocyte complexes from later stages of follicles, in contrast to the earlier stages of ovary development here. Alternatively, the concentration of melatonin used in this study may have been too low to protect against the concentration of B[a]P used. Finally, melatonin may have been ineffective as a protectant because B[a]P did not lead to an increase in oxidative stress, with the observed death of follicles due to the activation of an alternative apoptotic pathway.

### Vulnerability of fetal ovary to PAHs

The developing fetus is considered to be particularly vulnerable and susceptible to the toxicological effects of PAHs (Whyatt & Perera 1995, Choi *et al.* 2006, Barr *et al.* 2007), up to 10 times more vulnerable than the mother to PAH-induced DNA damage (Perera *et al.* 2005). This increased vulnerability might be partly due to the fetus having a decreased immune response and being less able to efficiently detoxify carcinogenic compounds than the mother (Anderson *et al.* 2000, Barr *et al.* 2007). Consequently, even minute amounts of xenobiotic chemicals can result in adverse developmental outcomes for the fetus (Crinnion 2009, Sexton *et al.* 2011). B[a]P-reactive metabolites have been found to accumulate in ovaries, with B[a]P-DNA adducts still being found in rat ovaries over 28 days following a single exposure to B[a]P (Ramesh *et al.* 2010). In the same study, concentrations of DNA adducts in the ovary were considerably higher than in the liver, suggesting that the ovary might be particularly vulnerable to B[a]P exposure.

The effects of maternal cigarette smoke can affect the fertility of female offspring, even when exposure is not during pregnancy (Sui *et al.* 2020). Female mice exposed to PAHs prior to mating and/or during lactation produced female offspring with up to a 70% reduction in the follicle pool, with lower quality oocytes (Jurisicova *et al.* 2007, Sui *et al.* 2020). Finally, levels of PAHs are higher in passive, second-hand smoke that is passed into the air, than that resulting from smoke inhaled directly into a smoker’s lung (Office on Smoking and Health 2006), resulting in the potential effect of continued exposure to the infant after birth.

## Conclusion

Given that the ovarian reserve is established during fetal life, any chemical such as B[a]P that can accumulate in the developing fetal ovary, affecting the development of germ cells and formation of ovarian follicles, could have long-term damaging consequences on the subsequent fertility of that individual. Results here support and expand on findings in previous work, demonstrating that B[a]P affects the formation and development of ovarian follicles in the developing mammalian ovary. When exposure occurs prior to the formation of the follicle, B[a]P reduces germ cell and subsequently PMF numbers. B[a]P exposure also causes DNA damage within mammalian germ cells and affects their progression through the early stages in prophase I of the first meiotic division. To the best of our knowledge, this is the first published report of this. B[a]P exposure after follicle formation affects the proliferative ability of the ovarian somatic cells and increases apoptotic cell death, leading to a reduction in the number of healthy follicles, primarily due to an effect on PMF numbers. There was no evidence of a significant increase in oxidative stress accompanying follicle loss, nor was there a protective effect of the antioxidant melatonin when co-administered with B[a]P.

In conclusion, the results here provide further evidence that the developing ovary is vulnerable to B[a]P exposure, both prior to and following PMF formation. Findings from this and other studies strongly suggest that fetal exposure to cigarette smoke, as well as other sources of B[a]P, could lead to reduced fecundity in adulthood.

## Supplementary Material

Supplementary figure 1. Total number and distribution of unhealthy follicles in embryonic (E13.5) and neonatal (PND4) mouse ovaries cultured with increasing concentrations of B[a]P. (A) Number and (B) distribution of ovarian follicles in embryonic ovaries classified as unhealthy following B[a]P treatment. (C) Number and (D) distribution of ovarian follicles in neonatal ovaries classified as unhealthy following B[a]P treatment. Bars denote mean + SEM. In the study on fetal ovaries (A, B) n = 7 for all treatment groups and n = 8 for control group. In the study on neonatal ovaries (C, D) n = 7 for 0.01 µg ml-1 and 0.01 µg ml-1 group, n = 9 for the 1 µg ml-1 group and n = 8 for control group. Stars denote significant differences relative to control. Stars denote significant differences relative to control (*p<0.05, **p<0.01).

## Declaration of interest

Norah Spears is Co-Editor-in-Chief of *Reproduction and Fertility* and was therefore not involved in the review or editorial process associated with this paper of which she is a co-author.

## Funding

This study did not receive any specific grant from any funding agency in the public, commercial or not-for-profit sector.

## Author contribution statement

AS: conceived, designed and coordinated the study, led experiments, analysed data, prepared figures and wrote the manuscript. MMar: contributed to experiments, analysed data and commented on earlier versions of the manuscript. MMat: contributed to experiments, analysed data and commented on earlier versions of the manuscript. CMA: contributed to experiments, analysed data and commented on earlier versions of the manuscript. NS: conceived, designed and coordinated the study and critically reviewed the manuscript. All authors read and approved the final version of the manuscript.
