# Supplementary material for: In vitro exposure to benzo[a]pyrene damages the developing mouse ovary
Source: Reprod Fertil. 2023 Apr 13;4(2):e220071. doi: 10.1530/RAF-22-0071 (PMC10160542; doi:10.1530/RAF-22-0071)

Supplementary materials

Supplementary figure 1. Total number and distribution of unhealthy follicles in embryonic (E13.5) and neonatal (PND4) mouse ovaries cultured with increasing concentrations of B[a]P. **(A)** Number and **(B)** distribution of ovarian follicles in embryonic ovaries classified as unhealthy following B[a]P treatment. **(C)** Number and **(D)** distribution of ovarian follicles in neonatal ovaries classified as unhealthy following B[a]P treatment. Bars denote mean + SEM. In the study on fetal ovaries (A, B) n = 7 for all treatment groups and n = 8 for control group. In the study on neonatal ovaries (C, D) n = 7 for 0.01 μg ml^{-1} and 0.01 μg ml^{-1} group, n = 9 for the 1 μg ml^{-1} group and n = 8 for control group. Stars denote significant differences relative to control. Stars denote significant differences relative to control (*p<0.05, **p<0.01).

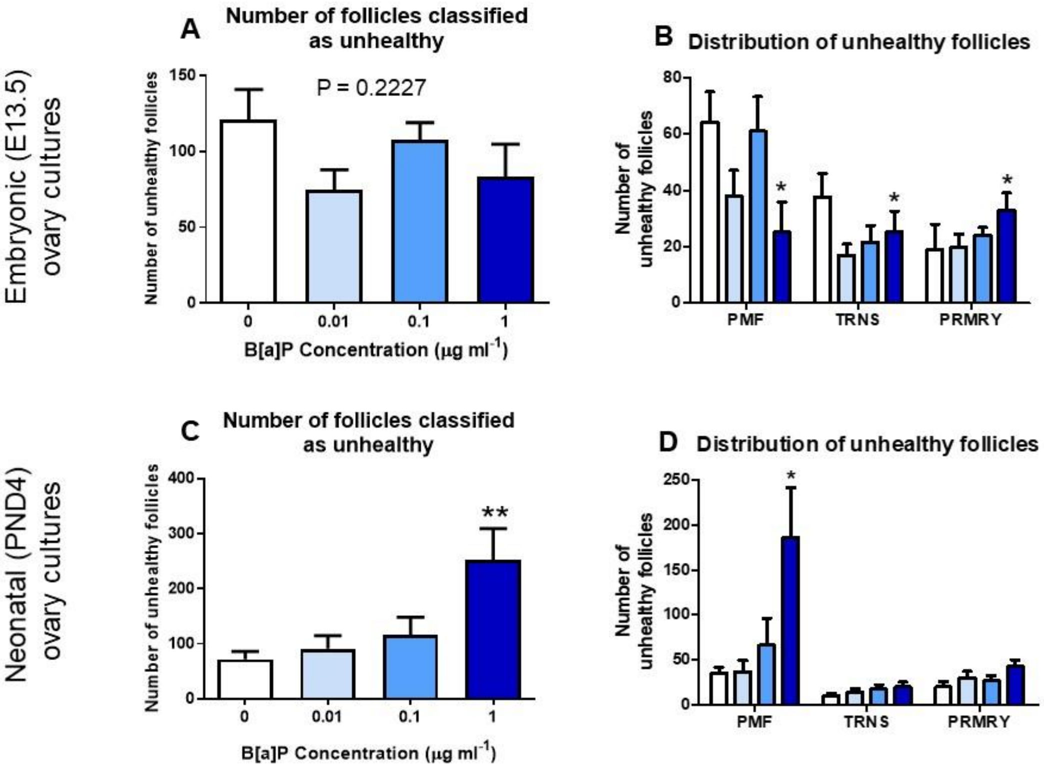

Supplement: Supplementary figure 1. Total number and distribution of unhealthy follicles in embryonic (E13.5) and neonatal (PND4) mouse ovaries cultured with increasing concentrations of B[a]P. (A) Number and (B) distribution of ovarian follicles in embryonic ovaries classified as unhealthy following B[a]P trea [file supplementary_figure_1.pdf]
